# Avalanche criticality in LaAlO$_3$ and the effect of aspect ratio

**DOI:** 10.1038/s41598-022-18390-7

**Published:** 2022-09-01

**Authors:** John J. R. Scott, Blai Casals, King-Fa Luo, Atta Haq, Davide Mariotti, Ekhard K. H. Salje, Miryam Arredondo

**Affiliations:** 1 School of Mathematics and Physics, Queen’s University Belfast, Belfast, BT7 1NN Northern Ireland, UK; 2 Department of Earth Sciences, University of Cambridge, Cambridge, CB2 3EQ England, UK; 3 School of Engineering, Ulster University, Jordanstown, BT37 0QB Northern Ireland, UK

**Keywords:** Materials science, Physics

## Abstract

Ferroic domain dynamics, as a function of external stimuli, can be collectively described as scale-invariant avalanches characterised by a critical exponent that are sensitive to the complexity of the domain microstructure. The understanding and manipulation of these avalanches lies at the heart of developing novel applications such as neuromorphic computing. Here we combine in situ heating optical observations and mean-field analysis to investigate the collective domain behaviour in pure-ferroelastic lanthanum aluminate (LaAlO$_3$) as a function of aspect ratio, the ratio of sample length to width, where the movement of the domains is predominantly driven by thermal stresses via thermal expansion/contraction during heat cycling. Our observations demonstrate that the aspect ratio induces (1) distinctive domain microstructures at room temperature, (2) a deviation of dynamical behaviour at high temperatures and (3) critical exponent mixing in the higher aspect ratio samples that accompanies this behaviour. While the critical exponents of each aspect ratio fall within mean-field predicted values, we highlight the effect that the aspect ratio has in inducing exponent mixing. Hence, furthering our understanding towards tuning and controlling avalanches which is crucial for fundamental and applied research.

## Introduction

Analogous to other ferroics (e.g. ferroelectrics and ferromagnetics), ferroelastics are characterised by regions of differently orientated states (domains), separated by domain walls, which form in order to minimise the free energy of the system and are switchable under the application of an external field^1^.

Domain switching has been at the forefront of fundamental and applied research, as it mediates many of the functional properties in advanced applications^2–5^. Ferroelastic switching is characterised by the movement of domain walls in small discrete jerks that emit long ranging elastic fields, which are likely to destabilise surrounding structures and trigger larger switching events known as avalanches, collectively constituting the typical ferroelastic stress - strain hysteresis^6^.

Particularly, the understanding of the physical mechanisms responsible for domain switching, in response to externally applied fields, is critical to the development of novel devices such as neuromorphic computing^7,8^; based on the jerky switching in materials with ferroelastic components^9–11^, which exhibits similar avalanche dynamics in neuronal activity^12^.

Mean-field theory has successfully characterised the collective movements of domains during switching events^11,13–20^, which predicts the power-law distribution of jerks as a scale-free process of the form $g(x)dx \sim x^{(-\varepsilon )} dx$^21^, whereby *g*(*x*) describes the probability (g) of a jump (*x*) and the energy exponent $(\varepsilon )$ is fundamental for classifying switching behaviours, taken from the maximum-likelihood method^16,22^. $\varepsilon$ can assume critical values under the appropriate circumstances such as 1.33 within mean-field theory. Such power-law critical exponents are scale invariant^23^ (with no characteristic size or time scales) and are independent of the microscopic properties of the system^24^ allowing for these systems to be characterised into different universal classes. This line of research has been used to determine universality amongst ferroelectrics^16^ and has demonstrated that the orientation of an applied field will influence the statistical behaviour of the system^25^.

As mentioned previously, characterising and controlling the response of domains (and domain walls) to external fields has been the main focus within the fields of domain dynamics and domain engineering; however, the system’s geometry is another aspect to device design which is currently poorly addressed when studying ferroelectric and ferroelastic domain dynamics. Indeed, previous reports have suggested that the domain microstructure can be altered due to the system’s elastic anisotropy^26^, while studies in smart-memory alloys^27^ have highlighted the effect of geometry on the material’s microstructure. Moreover, changes in the resultant domain patterning as a function of the aspect ratio have been shown in freestanding BaTiO$_3$^28,29^. However, systematic studies of ferroelectric and ferroelastic domain dynamics as a function of the aspect ratio are still lacking. Theoretical models for ferromagnets have elucidated how changing boundary conditions can influence the resultant domain configuration in samples with a particular focus on thickness effects^30^, but these models in part neglect switching behaviours.

Further to this, studies examining the dynamic behaviour in relation to critical exponent values, and its potential for mixing, as a function of macroscopic parameters, are currently less prominent despite the importance of these factors being highlighted in recent literature^14,17,21,22,31–33^.

In this study, in situ heating and optical microscopy are utilised in conjunction with mean-field statistical analysis to characterise the motion of ferroelastic domains (and domain walls) in LaAlO$_3$ as a function of thermally induced stress and geometric aspect ratio. The heat cycle causes thermal expansion/contraction^34,35^ of the sample, inducing thermal stresses^36^ which can arise from surface tensions and dead layers^29^, that effectively manifest as boundary conditions that drive the domain microstructure. Such stresses originate in the anisotropic nature of the crystal structure itself (Rhombohedral R-3c)^37^ which shows pronounced elastic anisotropy^38^ and are known to be influenced by temperature^39,40^.

We determine that stress anisotropy, induced by the sample’ aspect ratio (length over width), not only affects the final domain microstructure but also induces distinct domain dynamics with characteristic critical exponent values. Moreover, we report on the mixing of the critical exponent between accepted mean-field values. Thus, demonstrating that the sample aspect ratio can be implemented as a simple and effective means to induce a change in domain dynamics.

## Results

### Static observations

**Figure 1:**
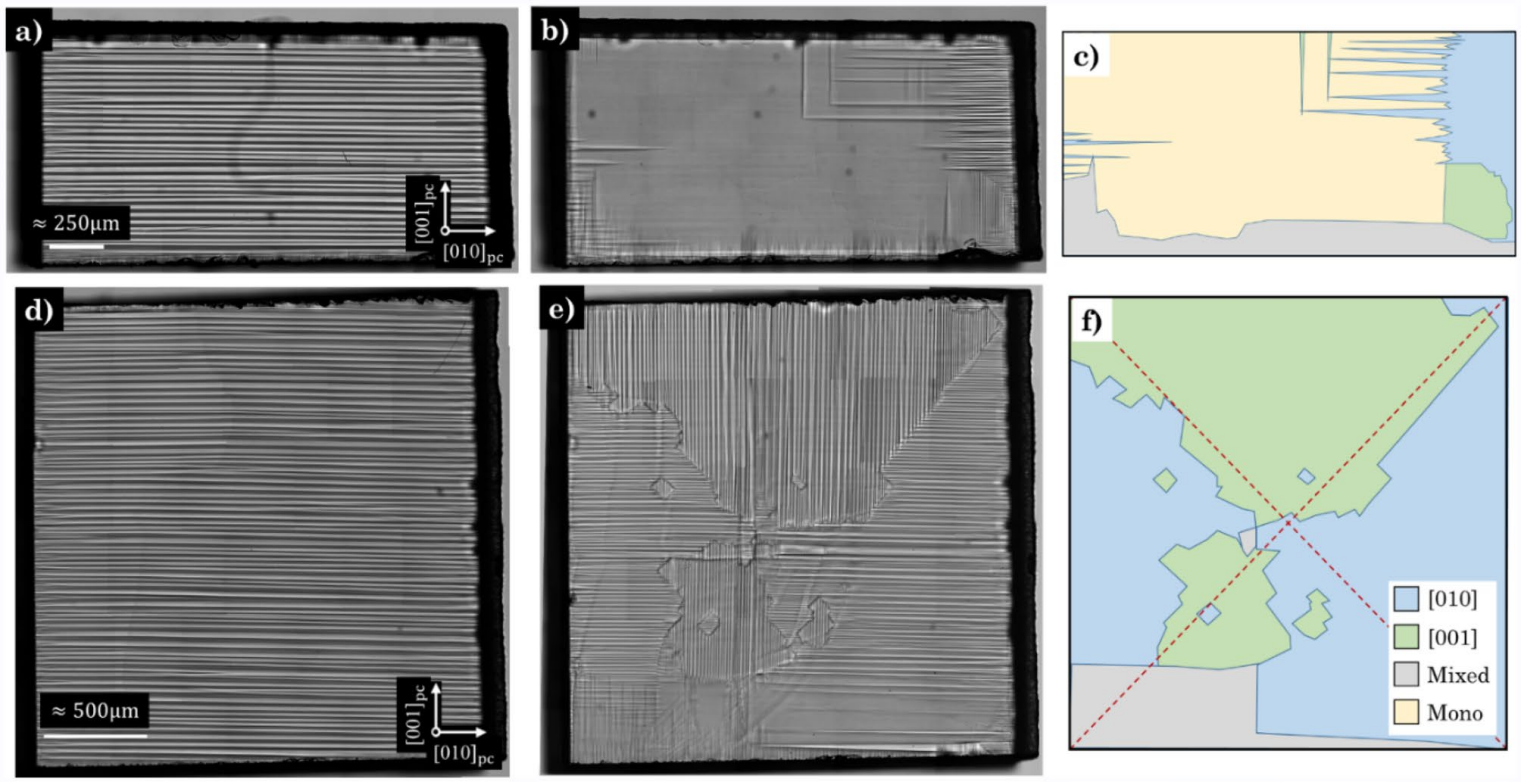
RT domain configuration pre and post annealing. Pristine samples (pre-annealing) of the (**a**) high aspect ratio ($\sim 1.8$) and (**d**) low aspect ratio ($\sim 1$) are compared against the post annealed samples after the third HC for (**b**) the high aspect ratio and (**e**) the low aspect ratio samples. (**c**) and (**f**) display the corresponding qualitative domain variant colour maps. The aspect ratio is calculated as the sample length ([010]_pc_) against width ([001]﻿_pc_).

In their pristine (pre-annealed) state, the two primary sample sets exhibited domains exclusively along the [010]$_\text {pc}$ (Figs. 1a and d). After the initial heat cycle (HC), each sample developed a different room temperature (RT) domain configuration, and importantly, a clear difference in the RT domain pattern was observed between the different aspect ratio sample sets.

In the case of the high aspect ratio sample set, a simpler RT domain configuration aligned mainly along the sample’s ‘long axes’ occurs, [010]$_\text {pc}$ (Fig. 1b and c), with fewer junctions and herringbone-like domains present (Supplementary Information S5), suggesting a different strain landscape to that of the low aspect ratio sample. It should be noted that due to resolution limits of the optical imaging technique employed (surface imaging), the regions where no obvious evidence of domain structure exists is referred to as mono domains, for simplicity. However, this does not negate the existence of sub surface domains or those nucleating from the opposite surface. Within these mono regions, transient domain patterning is observable, patterns resembling sub surface domain structures that persist well beyond the critical temperature of LaAlO$_3$ and back, which regularly manifest as similar phenomenon cited in literature^41,42^. Areas of mixed domain structures refer to the population of the two main domain variants ([010]$_\text {pc}$ and [001]$_\text {pc}$).

Comparatively, the low aspect ratio sample’s microstructure is divided roughly into densely populated quadrants (Fig. 1e and f) composed of the two predominant variants: [010]$_\text {pc}$ and [001]$_\text {pc}$, that meet approximately at the centre of the sample. Similar quadrant patterning has been observed in literature as closed platelets^28^ and by birefringence imaging^43^. Domain junctions are a dominant feature throughout this sample and form the boundaries of these quadrants, which increase in density towards the sample epicentre (Supplementary Information S5). Along these junctions period doubling can be observed, accommodating the thermal stresses of mutually exclusive symmetry variants that have not been able to relax into needle twins^44^. Further to this, a high population of herringbone-like domains^45^ exist throughout the sample that overlap with the bulk microstructure (Supplementary Information S6).

The effect that subsequent HCs have on the domain configuration at RT is further detailed in Supplementary Information S4, supported by the insights given by XPS (Supplementary Information S3). From observations given in the supplementary, there are some slight changes in the domain configuration as a function of HC, however, the more dominant effect is observed between samples with different aspect ratio.

Although implications of sample aspect ratio are touched upon in literature, they are not fully explored. For example, birefringence measurements conducted on samples of varying geometries have indicated an orientational preference for higher aspect ratio samples, or cross-hatch patterns in lower aspect ratio samples^43,46^. Perhaps the more direct evidence is given in the studies imaging free-standing BaTiO$_3$ via transmission electron microscopy^28,29^, that considers Landau formalism as an explanation for the changes of microstructural patterning at RT.

**Figure 2:**
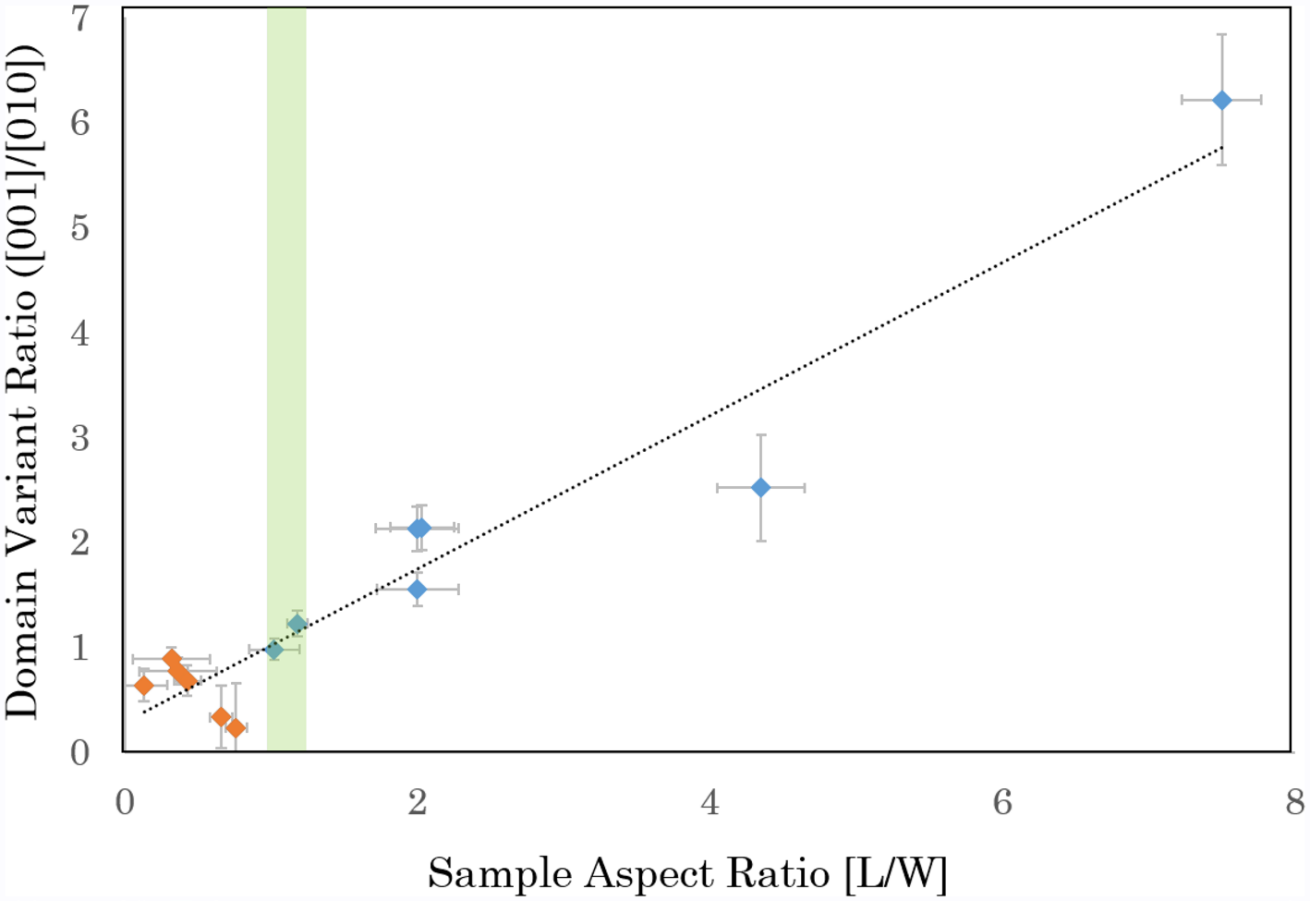
The variation of the domain ratio ([001]$_\text {pc}$ over [010]$_\text {pc}$) as a function of the sample’s aspect ratio, at RT, after HC. The samples’ aspect ratio, calculated as the sample length (L) against width (W), ranges from $\approx 7.5$ to $\approx 0.13$. Points in blue correspond to an aspect ratio greater than or equal to 1 and those in orange correspond to a lower aspect ratio. The region highlighted in green indicates the aspect ratio range in which patterning of the two domain variants are more equivalent.

In order to test this more rigorously, 13 additional samples were cut of the same bulk and heat cycled in an identical manner described in the experimental section (Supplementary Information S2). Each sample was then cut in two, and this process was repeated until a much lower aspect ratio was achieved, spanning from $\approx 7.5$ to $\approx 0.13$. It is important to mention that, as in the case of Fig. 1a and b, the domain structure ran parallel to the long axis of these samples in their pristine state, corresponding to the [001]$_\text {pc}$. Figure 2 shows the data from all samples investigated here, highlighting the effect of reducing the sample's aspect ratio in relation to the prevailing domain variant ([001]$_\text {pc}$/[010]$_\text {pc}$), where a general linear fit can be identified. For reference, areas indicated as mono regions have been excluded from these calculations as the incorporation of mixed domains and the [001]$_\text {pc}$ and [010]$_\text {pc}$ better represent the anisotropy that arises from the two different sample aspect ratios.

In general, three regions of interest can be inferred from the observations in Fig. 2. (1) Aspect ratios >1 represent a case where the length exceeds width, resulting in a similar scenario to that observed in Figure 1b where the [010]$_\text {pc}$ variant dominates. (2) Decreasing the aspect ratio until the length is roughly equivalent to the width, as highlighted within the green region of Fig. 2, which results in a more even split of domain variants similar to that of Fig. 1e. (3) Further decrease to the aspect ratio until the length is shorter than the width of the sample, whereupon the preference in domain variant switches to [001]$_\text {pc}$ displaying similar domain patterns observed in other high aspect ratio cases (Fig. 1b), supporting the concept that the aspect ratio greatly influences the resulting domain patterning at RT.

Importantly, the effect that the aspect ratio may impose in ferroelastic domain dynamics is not yet explored. Thus, we continue by investigating and characterising the domain dynamics for two aspect ratio sets.

### Dynamic observations

All samples were heated individually up to 600±5ºC ramping through the critical temperature (T$_\text {C} \sim$ 545ºC), the transition point between the rhombohedral ferrophase and cubic paraphase, and cooled down to RT at a variable rate (Supplementary Information S2). The dynamical behaviours during the ramp up stage were comparable between the two main sample sets (Supplementary Information S7).

The similarities in dynamic behaviour between aspect ratios continues past T$_\text {C}$ into the early stages of the the ramp down. A precursor pattern comprised of a numerous nucleation events occurs without preferential orientation (Supplementary Information S7), in a 20 ± 5ºC window below T$_\text {C}$. As the samples continue to cool down, coarser and more complex structures arise (Supporting Videos SV1–SV6), in which perpendicular domain variants begin to retract and become constrained to a host domain, forming mother-daughter kinks (Supporting Information S8). These kinks demonstrate the same scaffolding effect in which herringbone-like domains nucleate onto, from $\sim$ 500 ± 5ºC downwards (Supplementary Videos SV7–SV8). Up to this temperature, the dynamical behaviour for both aspect ratios is almost hysteric, with similar structures forming in the same regions and at the same temperatures, within each of the aspect ratio samples. It is only below this temperature that the dynamics, and consequent domain configuration, begin to greatly diverge.

**Figure 3:**
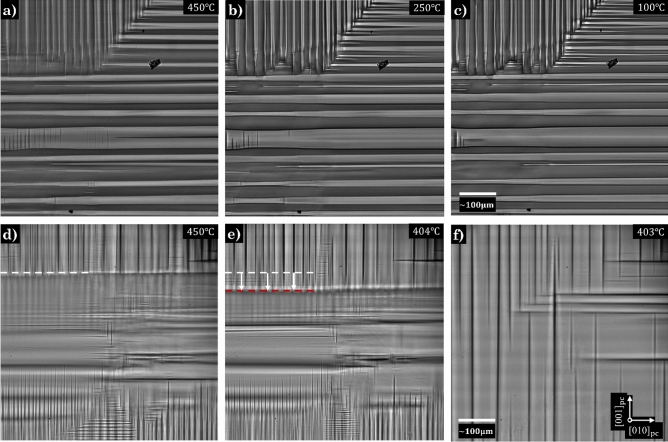
Domain dynamics during the cooling down stage. Domain structure for the low aspect ratio sample at (**a**) 450±5ºC, (**b**) 250±5ºC and (**c**) 100±5ºC, revealing a stable domain configuration that remains largely unchanged during the cooling sequence in which the high temperature patterning resembles the RT domain configuration. The high aspect ratio sample is displayed (**d**) at 450±5ºC showing the formation of the [010]_pc_ domain front that occurs at high temperatures (white dashed line), (**e**) domain front propagation in the [001]_pc_ direction (change in position marked by the white arrows and red dashed line) and (**f**) at 403±5ºC, just after the global reconfiguration occurs.

The low aspect ratio sample begins to adopt a similar configuration to that of its RT microstructure (Fig. 3a) from 525±5ºC, just after the precursor window below T$_\text {C}$, and remains largely unchanged throughout the HC (Fig. 3b and c). It is proposed that the more symmetric nature of the sample reinforces a more equal competition between variants dynamically, resulting in multiple junctions and high local stresses that hold a near stable configuration, in which internal friction^47^, domain jamming^48^ and pinning, increasingly take over as the dominant effects as the freezing regime^49^ is approached ($\sim$200°C).

Comparatively, below a 20±5ºC window from T$_\text {C}$, the high aspect ratio sample forms a domain front parallel to [010]$_\text {pc}$ (Fig. 3d) which becomes more mobile with decreasing temperature, propagating along the [001]$_\text {pc}$ direction (Fig. 3e). The domain front continues to propagate until a critical point is reached, and a global reconfiguration of the domain structure occurs (Fig. 3f), resulting in a less complex and sparse domain pattern, similar to the RT domain configuration (Supplementary Videos SV1–SV3). Additionally, it is worth noting that the temperature at which this reconfiguration occurs at, increases with the number of HCs, which could be attributed to the change in the population of oxygen vacancies (Supplementary Information S9), but would require further investigation.

To further understand and characterise the dynamics in both aspect ratio samples (for the cooling down stage), several spatiotemporal maps showing the avalanche activity as a projection along the x and y axes, as a function of temperature were produced.

**Figure 4:**
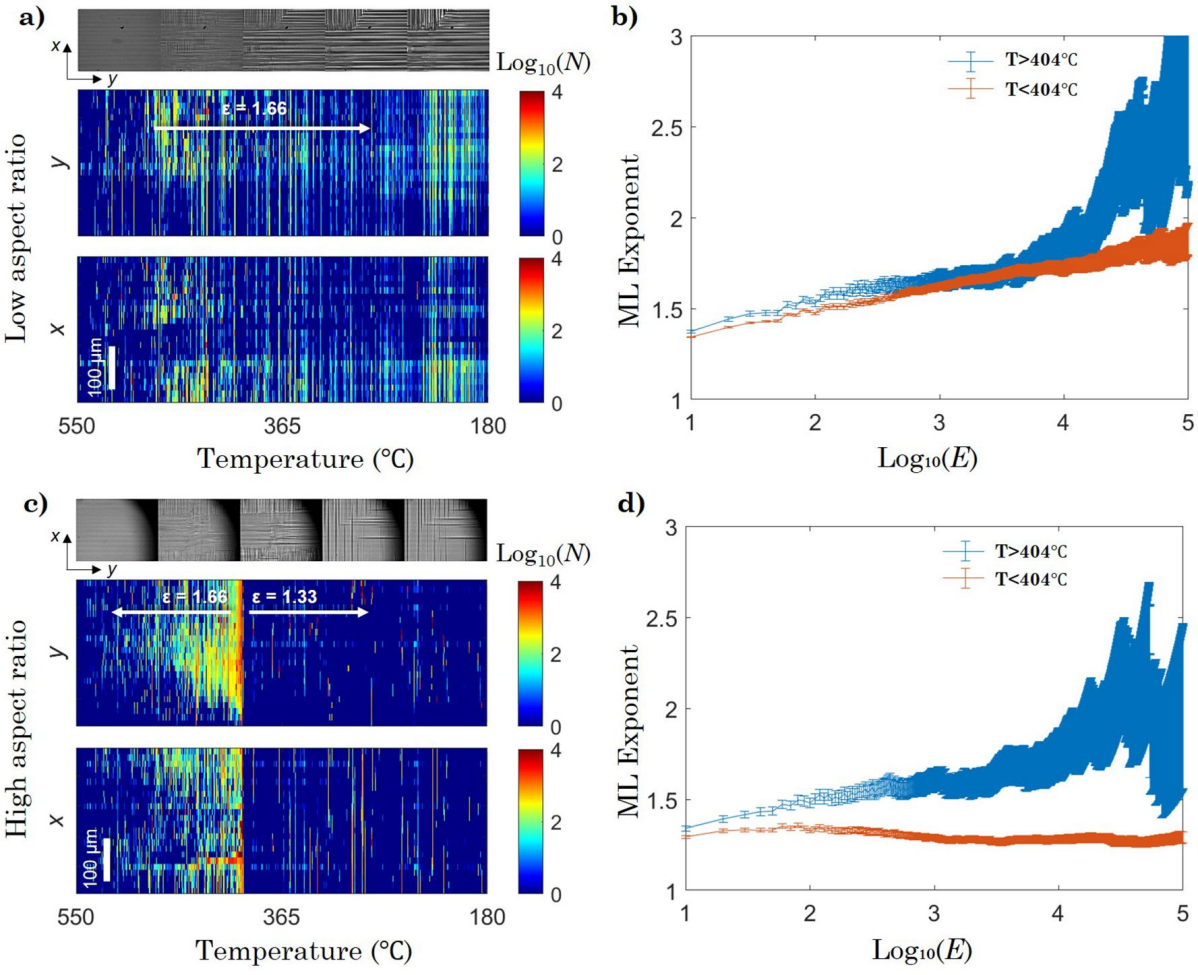
Spatiotemporal evolution of avalanche activity and power-law distribution. Spatiotemporal maps of avalanche activity as a function of temperature and corresponding optical progressions are shown for the (**a**) low aspect ratio sample and (**c**) high aspect ratio sample. The maximum likelihood (ML) exponent is derived the range of energies that plateau and follow a ower-law behaviour in the (**b**) low aspect ratio sample and (**d**) high aspect ratio sample, which shows a deviation from the value below 404±5ºC corresponding to a mixing of the energy critical exponent. The error bars of the exponents plotted in b) (±0.05) and d) (±0.15) correspond to the mean values of the error bars in the range where the ML exponent analysis exhibits a plateau (flattest range). The shadowing of the snapshots in c) are from an overlapping aperture.

The x axis corresponds to [010]$_\text {pc}$ and the y axis to the [001]$_\text {pc}$ (Fig. 4a and c). This was computed as the number of avalanches along x profiles drawn per each y, as well as the y profiles drawn per each x. Here (N) is activity, the number of areas changed between two consecutive frames which are defined in Eq. 1 of the experimental methods. As previously mentioned, both samples show similar behaviour at high temperatures, where activity gradually increases with decreasing temperature, however there are certain important differences: in the low aspect ratio sample, the activity is much lower and more evenly distributed across the x and y projections, which remains stable up to 180±5ºC (Fig. 4a). It is at this point that the behaviours of the freezing regime dominate, and the activity greatly decreases, thus no statistical analysis was performed for lower temperatures in both samples.

In the high aspect ratio sample, the activity increases from the centre of the field of view (long rectangular side) and from the short sides of the field of view (where vertical and horizontal needles intersect) up to a large rate peak ($10^{4}$ events/ºC) around 404±5ºC (Fig. 4c). Afterwards, the activity dramatically and suddenly reduces and then remains stable until $\sim$180±5ºC which are both consistent with the in situ optical observations made for the primary sample sets.

The avalanche distribution before and after the activity peak (global reconfiguration) in the high aspect ratio sample was further analysed. In the range of energies (E=A$^2$) in which the ML exponent is flat (plateau), a power law behaviour is demonstrated and a value for the exponent can be estimated. The power-law exponent was found to be $\varepsilon$ = 1.66±0.15 above 404±5ºC, and $\varepsilon$ = 1.33±0.05 below 404±5ºC (Fig. 4d) corresponding to the dramatic change in domain configuration seen in Fig. 3e and f. It should be noted that this reconfiguration experiences a temperature shift per HC (Supplementary Information S9). The same analysis however, reveals that the low aspect ratio sample exponent, $\varepsilon$ = 1.66±0.15, remains constant during the full cooling stage (Fig. 4b). These mean-field values are typical^18^, representing the energy distribution right at the critical point ($\varepsilon$ = 1.33) and stress-integrated energy distribution( $\varepsilon$ = 1.66) as described by Salje and Dahmen^13^, that stems from two effects; the local movement of pinned domain walls and the large interactions that come from the intersection of domain walls with one another^14^. For example, such values are also observed in smart-memory alloys^50,51^ and the collapse of porous materials^52,53^. Importantly, the difference in exponent for the high aspect ratio sample indicates a change of the ferroelastic dynamics, from one fixed point value to another.

These results confirm the effect that the aspect ratio has on the domain dynamics, as shown in the two aspect ratio sets here investigated, and it demonstrates a difference in the thermally driven stress anisotropy in regards to the distribution of activity between aspect ratios.

Although such exponent mixing is widely present in literature^14,17,21,22,31–33^, to our knowledge, this is the first direct experimental observation in a pure ferroelastic system that correlates a change in domain dynamics and critical exponent values, to the tuning of sample aspect ratio. It demonstrates that a change in fixed point values occurs for power-law distribution^22^ (critical exponent) and thus a change in characteristic behaviour occurs. These results were shown to be repeatable and have been reproduced in other sample sets of analogous aspect ratios taken from the same bulk LaAlO$_3$ sample (Supplementary Information S10 and Supplementary Video SV9).

The aspect ratio range, at which the change occurs between dynamical behaviour and subsequent critical fixed point values, can also be inferred from the regions identified in Fig. 2 for the RT microstructure. The green region in Fig. 2 indicates an even split in the domain variants and exhibits the more stable dynamic behaviour, where a domain configuration resembling that of the RT pattern (during the cool down stage) is adopted at a high temperature. At either side of this range, a strong preference to domain variants is given and thus the thermal stresses imposed by the exaggerated aspect ratio causes distinct dynamic behaviour in which large cascades and possible re-configurations occur. Typically, this will result in a domain pattern that is primarily aligned with the long axis of the sample although this is more readily seen above an aspect ratio of 1 where considerations to sample size effects are less prevalent.

### Discussion

Ferroelastic domain walls propagate as jerks that emit strain fields^15^, which have the ability to de-stabilise other domain walls, precipitating more jerks and thus creating avalanches. As these elastic interactions are non-local, avalanches demonstrate a logarithmic shape-dependency classically^26^ and can be characterised by a critical exponent value $\varepsilon$.

In this work we use in situ heating combined with in-plane optical microscopy to induce thermal stresses, as the driving force behind domain wall motion, and provide experimental evidence to demonstrate that tuning the aspect ratio of pure-ferroelastic LaAlO$_3$ influences not only the resultant domain structure at RT, but also its dynamics. The resultant RT domain patterning can be complicated due to the relatively long range of elastic forces. This materialises as a strong anisotropy in the RT patterning along the ‘long’ axis of the high aspect ratio sample, in comparison to the low aspect ratio sample, which formed quadrants of the predominant variants of the $<100>_\text {pc}$, resulting in what appears to be a more uniform strain on the sample enforced by the more symmetric boundary conditions as a consequence of the sample's more symmetric geometry . Broadly, this emerges as a linear dependency between domain variant preference and aspect ratio.

Moreover, we report on the distinct domain dynamics between the samples: the lower aspect ratio sample displayed a microstructure that resembled its RT pattern at high temperatures, while the high aspect ratio sample demonstrated a global reconfiguration of the microstructure that was premediated by a large cascading domain wall front, which develops into a sparser and less complex configuration that slowly relaxed into its RT patterning.

Importantly, these in situ optical observations were combined with a statistical analysis by mean-field theory to calculate the critical exponent values and characterise the behaviour observed in each aspect ratio. This exhibited an additional anisotropy in domain activity along the long x-axis of the sample. In the low aspect ratio sample, the field integrated critical value of $\varepsilon$=1.66±0.15 held throughout the entirety of the cool down stage. On the other hand, the high aspect ratio sample exhibited a change in fixed point critical exponent value at the critical point, from $\varepsilon$=1.66±0.15 to $\varepsilon$=1.33±0.05, which corresponded to the global reconfiguration and variation in dynamical behaviour, commonly referred to as exponent mixing. These critical exponent values bear statistical similarities to those reported for other ferroic systems^14,16,18,54^, notwithstanding other studies on LaAlO$_3$^55,56^, validating a universality amongst such materials, although similar dynamical observations appear lacking in ferroelectric systems. This study, to our knowledge, is the first to directly address the correlation between the sample’s aspect ratio, domain structure and critical exponent mixing. We suggest more studies on ferroic systems could benefit from utilising a similar approach to identify such behaviours, which may otherwise be overlooked^16,18,57^, and is critical to further our understanding of ferroic dynamical behaviour and development of future smart devices.

### Experimental methods

LaAlO$_3$ was selected as the archetypal sample as it is a well-documented pure-ferroelastic^58–60^, as well as being translucent and chemically stable, making it an ideal choice for optical studies^3^.

15 samples were tested in total, ranging from an aspect ratio of $\approx 7.5$ to 0.13, with a focus given to two sample sets testing two differing aspect ratios (length against width): i) square sample with a low aspect ratio of 1:1 ($\approx [2.25, 2.25, 0.5] \pm 0.25$mm) and ii) a rectangular sample with a higher aspect ratio of $\sim 1.8(\approx [2.25, 1.25, 0.5] \pm 0.25$mm).

All samples were cut from the same bulk LaAlO$_3$ (MTI corp.), therefore minimising differences in chemical composition, with a diamond wire saw optimised at a slow cutting setting to reduce imposed mechanical stress. Samples were cleaned with acetone prior to imaging.

The optical set up utilised bright-field, transmission imaging with a 10x objective lens (Supplementary Information S1). Static images of the samples were acquired before and after each heat cycle (HC) utilising an in-plane imaging technique with the polariser set at 45º to the [010]$_\text {pc}$ crystallographic direction, in order to maximise the contrast between the symmetry equivalent domains of the same variant^61^.

A cross-polar set up was additionally implemented for comparison. In situ recordings were taken using in-plane imaging as domain dynamics were more discernible around the critical temperature (T$_\text {C}$ $\sim \text {545C}$).

The samples were thermally cycled using a transmission-mode heating chamber (Linkam) in the light path of the optical set-up (Supplementary Information S1). All samples were heated from room temperature (RT) to 600±5°C at the same variable rate which was mirrored in the ramp down to RT (Supplementary Information S2). Samples that had undergone the same number of heat cycle iterations were compared in order to minimise any chemical differences between sample sets. Heating was conducted in an isolated sealed chamber under atmosphere with each sample undergoing several HCs. It should be noted that each HC was stopped just below 50ºC due to technical restrictions.

X-ray photoelectron spectroscopy (XPS) was carried out to analyse changes in the chemical composition at the surface of the sample ($\sim$10 - 20nm), as a function of HC (Supplementary Information S3). The analysis revealed that overall, there is a decrease of oxygen vacancies with heat cycling, as expected within a reducing environment. Another indicator of the change in oxygen content is the colour of the sample^62^. The pristine sample is translucent with a blue tinge^63^ and after several HCs, the samples acquire a brown tinge, further confirming the increase of oxygen whilst annealing in atmospheric conditions. This change in colour is not representative in the figures within this document.

The domain dynamics were investigated using acquired in situ videos of roughly the same area in each HC, with a field of view $\sim$800x600$\mu$m. These areas are identified using a pixel by pixel analysis^22^ on the in situ videos based on the difference between consecutive frames ($\triangle$t = 1 s). Firstly, pixel by pixel, the intensity of jerks (J$_{\text {ij}}$) as a function of time are defined by the Eq. 1.

1 $$\begin{aligned} \mathrm {J}_{\mathrm {ij}} = \left( \frac{\mathrm {dB}_{\mathrm {ij}}}{\mathrm {dt}}\right) ^2 \end{aligned}$$

where B$_{\text {ij}}$ is the intensity at each pixel with spatial coordinates i and j. Then, the areas are defined by the connected pixels (J$_{\text {ij}}$) larger than a threshold, which is twice the mean value of the pixel’s noise. Each area (A) is considered as an individual event and its distribution of sizes follows a power-law, a key feature for avalanche dynamics. The energy associated at each area is defined by E = A$^2$, where the energy exponent ($\varepsilon$) is used as it is the common magnitude for universality comparisons.

In order to estimate the power-law exponent ($\varepsilon$) for these energies, the probability density g(E) which outlines the avalanche events’ distribution of energies, follows a power-law behaviour at criticality;

2 $$\begin{aligned} \mathrm {g}(\mathrm {E})\mathrm {dE} = (\varepsilon -1) \left( \frac{\mathrm {E}}{\mathrm {E}_{\mathrm {Min}}}\right) ^{-\varepsilon } \frac{\mathrm {dE}}{\mathrm {E}_{\mathrm {Min}}} \end{aligned}$$

in which energy exponents vary typically between 1.33 and 2.5, bar some reports of values exceeding this^22^.

This can then be fitted by the maximum likelihood method^64^, in which a series of measured values (E$_k$) with k = 1, 2$\ldots$, N omits data below a changeable cut-off (E$_0$), which when fixed, gives an estimation of the energy exponent of^50,65^;

3 $$\begin{aligned} \varepsilon = 1+\mathrm {N}_{\mathrm {E} \geqslant \mathrm {E}_0} \left[ \sum _{\text {i=1}}^{\mathrm {N}_{\mathrm {E} \geqslant \mathrm {E}_0}} \ln \left( \frac{\mathrm {E}}{\mathrm {E}_0}\right) \right] \end{aligned}$$

where ${\mathrm {N}}_{(\mathrm {E} \geqslant \mathrm {E}_0)}$ is the number of events equal or greater than E$_0$.

## Supplementary Information

Supplementary Information.

Supplementary Video 1.

Supplementary Video 2.

Supplementary Video 3.

Supplementary Video 4.

Supplementary Video 5.

Supplementary Video 6.

Supplementary Video 7.

Supplementary Video 8.

Supplementary Video 9.

## Data Availability

The data that support the findings of this study are available from the corresponding author upon reasonable request.
