# Supplementary material for: Avalanche criticality in LaAlO3 and the effect of aspect ratio
Source: Sci Rep. 2022 Sep 1;12:14818. doi: 10.1038/s41598-022-18390-7 (PMC9437108; doi:10.1038/s41598-022-18390-7)
Supplement: Supplementary file 1 — Supplementary Information. [file 41598_2022_18390_MOESM1_ESM.pdf]

Supplementary Information; Avalanche Criticality in $LaAlO_3$ and the Effect of Aspect Ratio

John J. R. Scott[1,*], Blai Casals[2], King-Fa Luo[1], Atta Haq[3], Davide Mariotti[3], Ekhard K. H. Salje[2], and Miryam Arredondo[1]

[1]Queen's University Belfast, Centre for Nanostructured Media, Belfast, BT7 1NN, Northern Ireland
[2]University of Cambridge, Department of Earth Sciences, Cambridge, CB2 3EQ, England
[3]Ulster University, School of Engineering, Jordanstown, BT37 0QB, Northern Ireland
[*]jscott63@qub.ac.uk

Supplementary Videos

Supporting video files of the ramp down stage of listed Heat Cycles (HC) are provided at 20x play speed utilising the experimental set up shown in Supplementary Figure S1. T_C of $LaAlO_3$ is $\sim$ 545°C. These can be found at [link] and are supplied as follows:

- **SV1)** High aspect ratio cooldown from T_C to 50±5°C for HC1
- **SV2)** High aspect ratio cooldown from T_C to 50±5°C for HC2
- **SV3)** High aspect ratio cooldown from T_C to 50±5°C for HC3
- **SV4)** Low aspect ratio cooldown from T_C to 50±5°C for HC1
- **SV4)** Low aspect ratio cooldown from T_C to 50±5°C for HC1
- **SV5)** Low aspect ratio cooldown from T_C to 50±5°C for HC2
- **SV6)** Low aspect ratio cooldown from T_C to 50±5°C for HC3
- **SV7)** Low aspect ratio kink and herringbone formation in HC1
- **SV8)** Low aspect ratio demonstration of mobile kinks in HC1
- **SV9)** High aspect ratio sample set 2 cooldown comparison in HC1

Supplementary Information and Graphics

Supplementary Information S1) Experimental Set-up

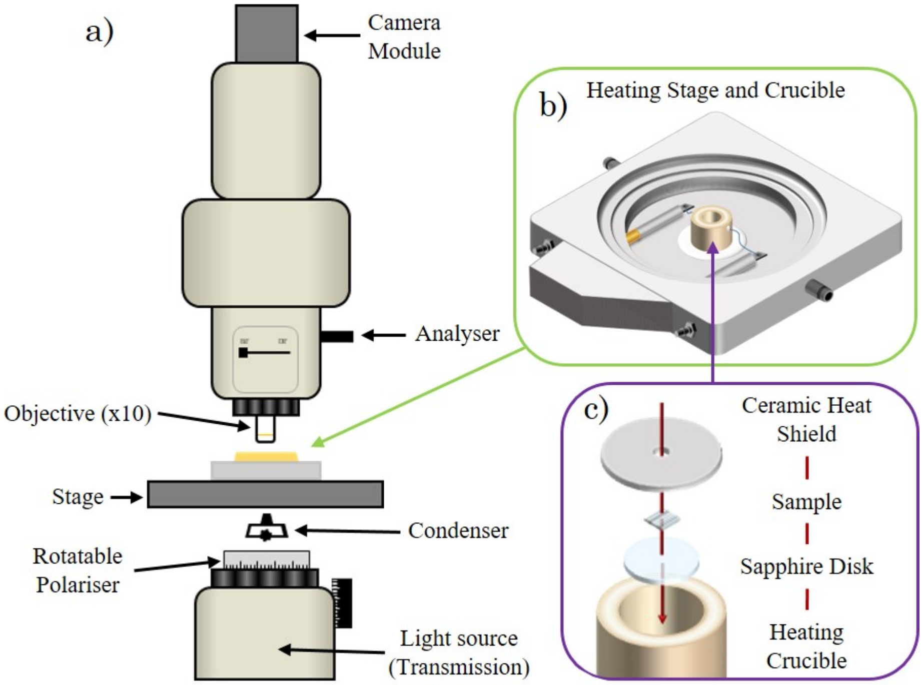

Figure S1. Overview of optical set-up and the heating stage. a) The optical microscope that employed bright-field transmission imaging with a 10x objective lens. For cross polar imaging the analyser was inserted and the rotatable polariser set to 90° to the crystals major axis. In-plane imaging required the analyser to be removed and the polariser set to 45° to the major axis of the sample. In-plane was selected as it allowed for clearer imaging of the domain structure around T_C but data was collected and compared with both techniques. This housed **b)** the heating stage that sat in the light path of the setup which was attached to a water pump for cooling and controlled heating and was sealed under atmosphere. The crucible housed **c)** the sample which was placed on a sapphire disk and then sealed with a heat shield that had an aperture to allow for transmission imaging.

Supplementary Information S2) Heat Cycle Conditions

Ramp Stage	Temperature Limit (°C)	Heat/Cool Rate (°C/min)
0	25	0
1	100	50
2	300	20
3	450	20
4	500	10
5	510	5
6	550	3
7	600	5
8	550	3
9	510	5
10	500	10
11	450	20
12	300	20
13	100	20
14	37.5	50

Figure S2. Heat cycle ramp overview. The stages of the heat cycle are provided which show the temperature the stage went up to (Temperature limit) and at what rate. This heat cycle procedure was implemented under atmosphere (air) for all samples investigated in this study. The cycle ended approximately 15-25°C higher than the starting temperature due to limits of the cooling system.

Supplementary Information S3) XPS Analysis

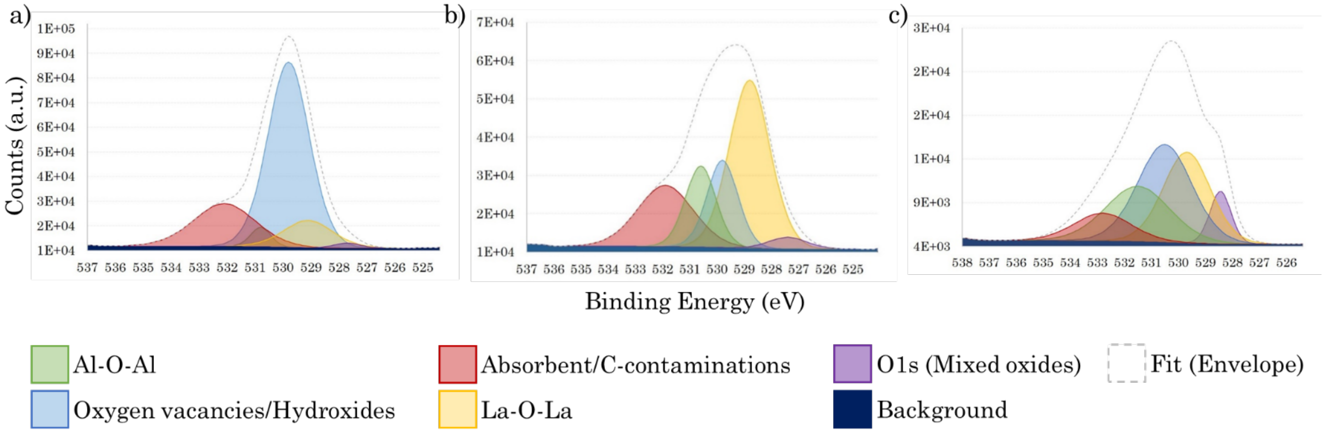

Figure S3. X-ray Photoelectron Spectroscopy data for HC tests. De-convoluted Peaks for O1s state shown for the **a)** pristine sample, **b)** sample after 1 HC and **c)** sample after 3 HCs. Notably the oxygen region of the XPS analysis shows an overall decrease in the number of oxygen vacancies, where the overall reduction is shown as a shift to the left of the oxygen vacancy peak. This can be affected additional by the presence of contaminants and carbon. The background has been partially removed for the figures.

Supplementary Information S4) Heat Cycle Effect on Domain Configuration

All samples were cut from the same as-bought bulk sample (also referred to as pristine) and annealed under the same conditions per experiment iteration. This was done in order to minimise the chemical differences between sample sets of different aspect ratio, to allow a more direct comparison after each heat cycle (HC). Furthermore, the effect that subsequent HCs have on the overall domain configuration was tested and minimum changes of the RT domain configuration were observed between HCs. Figure S4 displays the overviews from before and after each HC at RT for the high and low aspect ratio samples.

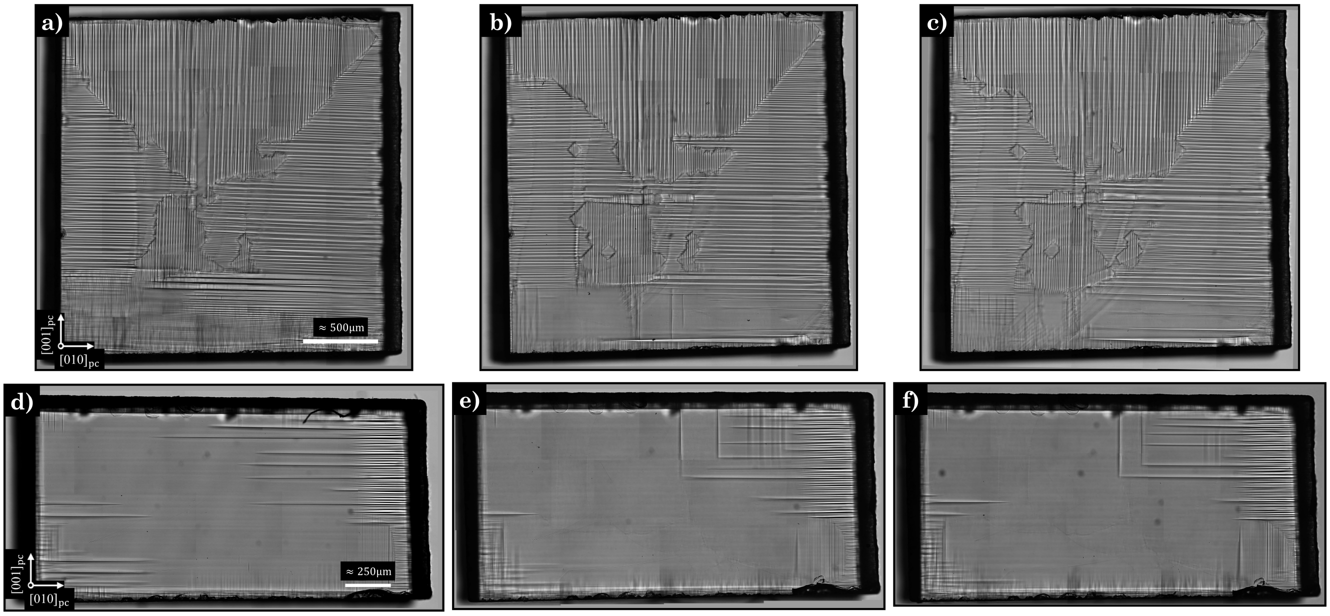

Figure S4. Overview at RT after each HC for the low and high aspect aspect ratio sample sets. The low aspect ratio sample after the **a)** first HC, **b)** second HC and **c)** third HC,as well as the overviews for the high aspect ratio samples after the **d)** first HC, **e)** second HC and **f)** third HC are provided.

For example, in the case of the low aspect ratio sample set illustrated in Supplementary figures S4 a-c, small islands of alternate domain variants arise, and the retraction of some larger features occur. The overall structure however, e.g. the main feature of quadrants of the two predominant domain variants, remains regardless of the HCs. In the high aspect ratio sample set (Supplementary Figures S4 d-f) there is some clear retraction and nucleation of domain variants between HCs but the and the alignment of the domains along the 'long' axis of the sample prevails. This examination might support the XPS observations (Supplementary Information S3). This is, the largest change in both domain structure and decrease in oxygen vacancies is observed after the first HC, and the changes observed with subsequent heat cycles is in comparison much smaller. The main difference in the domain structure is observed between samples with different aspect ratios and not as a function HC. This strongly suggests that the effect of annealing (and associated chemical changes) is comparable between samples that have undergone the same number of heat cycles and it suggests that the aspect ratio has a more dominating effect in altering the domain structure.

Supplementary Information S5) Density of Domain Structures

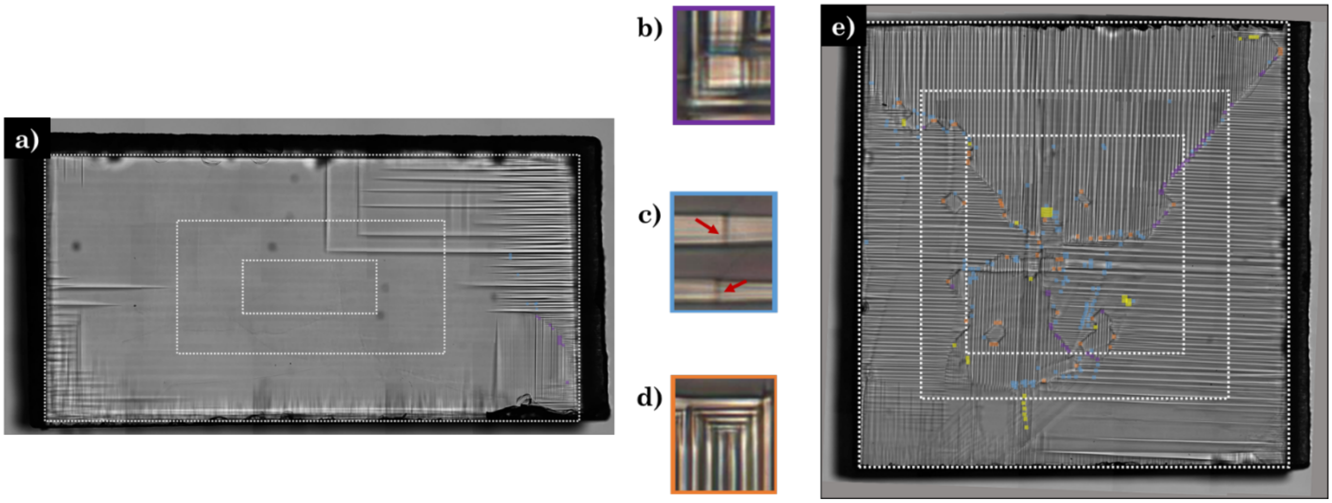

Figure S5. Density maps of domain structures. a) The high aspect ratio sample which exhibits few of the structures shown as **b)** 'box junction' **c)** kink domain **d)** 'temple domain'. Alternatively, in **e)** the low aspect ratio sample, these structures exist throughout the sample and increase with density towards the centre of the sample suggesting the strain in the sample also increases in the same manner.

Supplementary Information S6) Herringbone-Like Domains

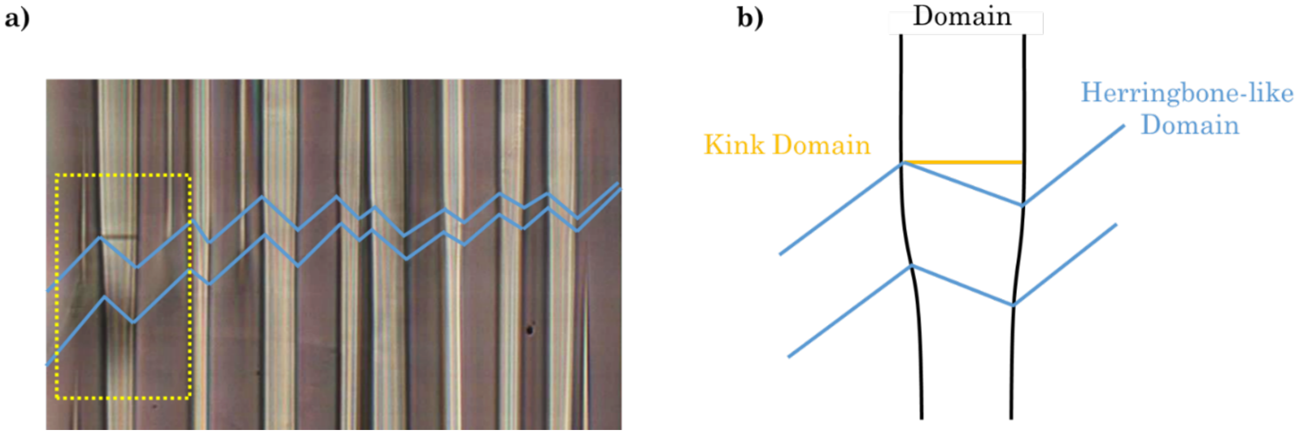

Figure S6. Kink domain and herringbone-like domain nucleation. a) Shows a micrograph of a domain that has nucleated a perpendicular variant from a kink in the domain wall. These kink domains appear to be the antecedent for structure such as the herringbone-like domains where some have pinned and remained at RT. This typically bends the intersecting domain to accommodate the local stress. This is shown schematically in **b)**. The intersection of herringbone-like domains with the bulk microstructure causes the bulk domains to bend and narrow; additionally, these exhibit a connecting perpendicular variant within the domain resembling the concept of super-domains described in the work of Salje et al.[1]. The bending of the domains has been previously reported as a typical elastic response of the system[2], which emerges as a wiggle of the interface and can be characterised by the Larkin length[3]. Another possible explanation for the narrowing is that the domain intersected by these herringbone-like structures, experiences both spontaneous expansion and contraction[4], wherein conjunction with pinning effects, creates bending in which a kink forms to compensate for the bending.

Supplementary Information S7) Ramp up to T_C Both samples showed similar behaviours during the heating up to T_C. Domain mobility was minor at temperatures as low as 100°C, corresponding to the freezing temperature regime. With the increase of thermal energy, domain walls begin to unpin and the ratio of static to mobile domains decreases between 250±5°C to 525±5°C, which is typical of the crossover to the superelastic temperature regime, characterised by a dramatic decrease of the Young's modulus[5] and internal friction between domains. Within this temperature range, similar structures nucleate in the same areas per heat cycle demonstrating a dynamic hysteric memory, as well as more mobile structures such as kink domains which are further described in the Supplementary Information S8. As T_C is approached (∼525±5°C), a high density of fine 'precursor' domains of $[010]_{pc}$ and $[001]_{pc}$ orientations nucleate without any apparent preference (Supplementary Videos SV1-6). This precursor pattern increases in density and coarsens as the temperature increases until annihilating at T_C, corroborated with a cross-polar set up. This behaviour has been briefly mentioned in literature[4], and it suggests that as the thermal energy increases, it may sufficiently reduce the energy barrier between twin orientations, allowing clusters of tilted octahedral to flip between the $< 011 >$, $< 111 >$ and $< 001 >$ axes[6]. It should be mentioned that the nucleation sites of these domains could be outside the field of view of the optical set up.

Supplementary Information S8) Kink Domains Within the superelastic regime, kink domains (Supplementary Video SV8) are a common feature of both samples and are now more clearly observed *in situ*. The kink in the domain wall nucleates a "domain in a domain"[1] that is readily mobile and can traverse sideways within the host domain[7] up to supersonic speeds[8]. During this movement, the kink domain readily splits into separate domains that move relative to one another as a daughter pair from a mother domain[2]. These kinks appear to act as a scaffold for the formation of greater hierarchal structures such as the herringbone-like domains, which nucleate onto these structures (Supplementary Videos SV7-8). It is suggested that this occurs as not only does the system need to address the immediate changes in local stress, but as a domain wall cannot simply 'end in-itself 'which would void the idea of defined polarity[9], these highly mobile kink domains become the antecedent to such structures that can create a closed polarity as well as accommodate higher stresses and maintain the average global shape of the sample. It is the need for defined polarity that needle domains exist (ends at another wall) or walls nucleate to the boundary of the sample[10,11]. With increasing temperature (range), a large number of these kink domains, grow beyond their initial confinement.

Supplementary Information S9) Temperature Shift of Observed Dynamics The temperature at which this global reconfiguration occurs at, shifts per heat cycle at 282±5°C,385±°C and 404±5°C respectively (Supplementary Videos SV1-3). In principle, this could be attributed to the reduction of oxygen vacancies that act as pinning sites and are capable of influencing the dynamics of the system. The concentration of these point defects are capable of affecting the bonding energy of the crystal[12]. Explicitly, this dependence increases the elastic modulus value with decreasing oxygen vacancy concentration which further impacts the thermo-mechanical properties of the system[13]. Previous work on other perovskite structures[14] has shown that the introduction/elimination of oxygen vacancies relaxes/compels the driving force behind the tilting of the otherwise rigid octahedra, where there is an evident change in the transition temperature as a function of oxygen vacancies[14]. Although the avalanche front and subsequent domain reconfiguration is not a phase transition of $LaAlO_3$, as no reduction in symmetry occurs, it could be that the change in oxygen vacancies have a similar effect and modify the temperature regime in which certain domain dynamics transpire, driven by the influence of the aspect ratio on the order parameter. This is not as clearly seen in the low aspect ratio sample, as it is proposed that the more equivalent energy landscape keeps the domain configuration in a metastable state and no significant change in the dynamics occur in order to note this effect and the effective anisotropy from defects is less prominent[12].

Supplementary Information S10) Statistical Analysis of Equivalent Aspect Ratios

Further testing of additional samples also concluded that starting off with a high aspect ratio and cutting the same sample to a low aspect ratio demonstrated the same statistics, similar to the process described in the static portion of this study.

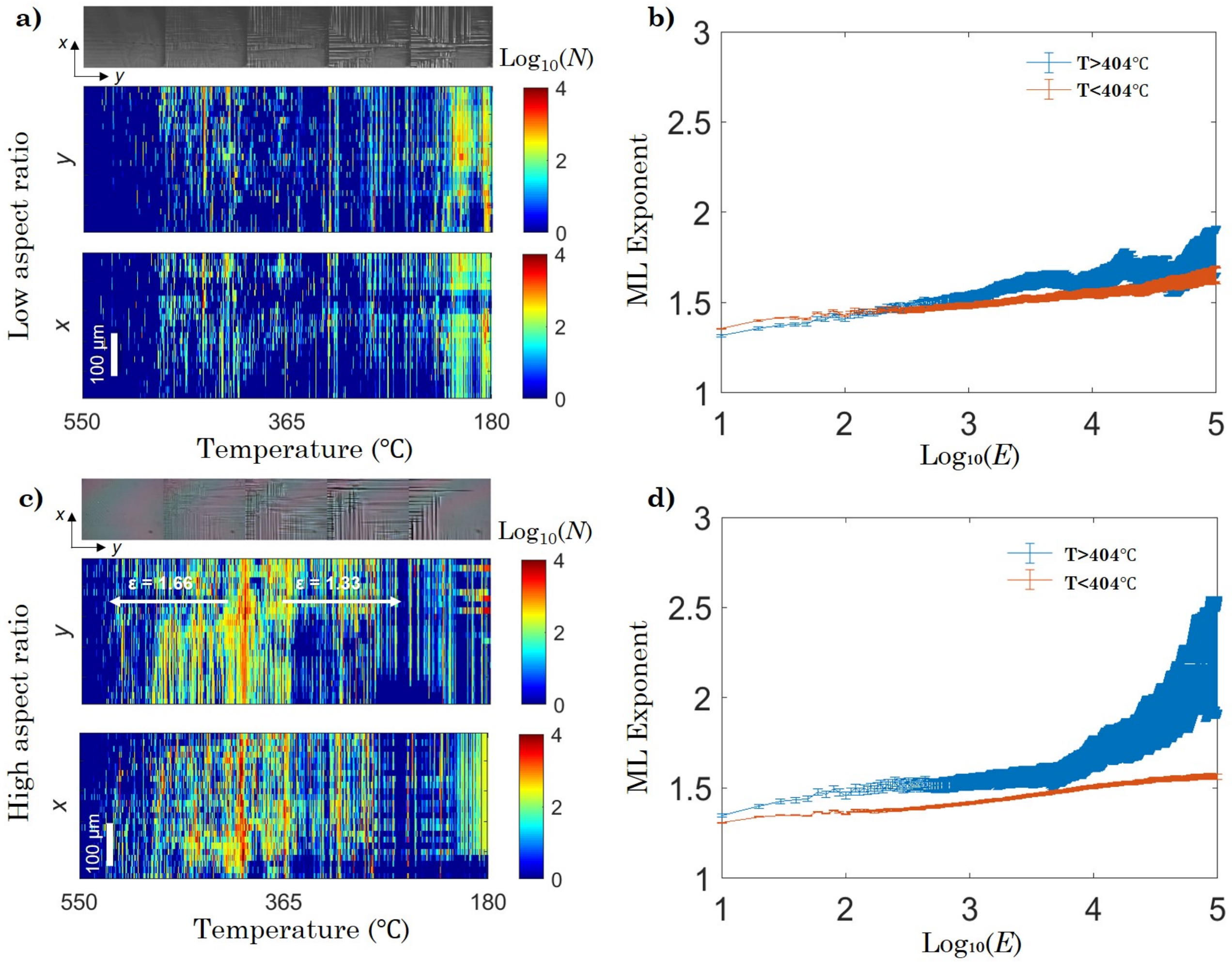

Figure S7. **Spatiotemporal evolution of avalanche activity and power-law distribution of second sample set.** Spatiotemporal maps of avalanche activity as a function of temperature and corresponding optical progressions are shown for the **a)** low aspect ratio sample and **c)** high aspect ratio sample. The maximum likelihood (ML) exponent is derived from the gradients in the **b)** low aspect ratio sample and **d)** high aspect ratio sample, which shows a deviation from the value below 404±5°C corresponding to a mixing of the energy critical exponent. This sample set demonstrates the same statistical behaviour as seen in the primary sample set and follows the same general behaviour, although activity is more pronounced after the critical exponent mixing (Supplementary Video SV9). The process of domain front formation, propagation and reconfiguration has been observed in other high aspect ratio samples and is available upon request.

References

1. Salje, E. K. H., Aktas, O., Carpenter, M. A., Laguta, V. V. & Scott, J. F. Domains within domains and walls within walls: Evidence for polar domains in cryogenic srtio3. *Phys. Rev. Lett.* **111**, DOI: 10.1103/physrevlett.111.247603 (2013).
2. Salje, E. K. H. Ferroelastic domain walls as templates for multiferroic devices. *J. Appl. Phys.* **128**, 164104, DOI: 10.1063/5.0029160 (2020).
3. Harrison, R. J. & Salje, E. K. H. Ferroic switching, avalanches, and the larkin length: Needle domains in $LaAlO_3$. *Appl. Phys. Lett.* **99**, 151915, DOI: 10.1063/1.3650475 (2011).

4. Harrison, R. J., Redfern, S. A. T., Buckley, A. & Salje, E. K. H. Application of real-time, stroboscopic x-ray diffraction with dynamical mechanical analysis to characterize the motion of ferroelastic domain walls. *J. Appl. Phys.* **95**, 1706–1717, DOI: 10.1063/1.1639949 (2004).
5. Mizzi, C. A., Guo, B. & Marks, L. D. Twin-boundary-mediated flexoelectricity in $LaAlO_3$. *Phys. Rev. Mater.* **5**, DOI: 10.1103/physrevmaterials.5.064406 (2021).
6. M A Carpenter, S. V. S. & Bass, J. D. Elastic relaxations associated with the Pm3 m-R3 c transition in $LaAlO_3$: II. mechanisms of static and dynamical softening. *J. Physics: Condens. Matter* **22**, 22, DOI: 10.1088/0953-8984/22 (2010).
7. Lu, G., Li, S., Ding, X., Sun, J. & Salje, E. K. H. Electrically driven ferroelastic domain walls, domain wall interactions, and moving needle domains. *Phys. Rev. Mater.* **3**, DOI: 10.1103/physrevmaterials.3.114405 (2019).
8. Salje, E. K. H., Wang, X., Ding, X. & Scott, J. F. Ultrafast switching in avalanche-driven ferroelectrics by supersonic kink movements. *Adv. Funct. Mater.* **27**, 1700367, DOI: 10.1002/adfm.201700367 (2017).
9. Sharma, P., Schoenherr, P. & Seidel, J. Functional ferroic domain walls for nanoelectronics. *Materials* **12**, 2927, DOI: 10.3390/ma12182927 (2019).
10. Lu, G., Li, S., Ding, X., Sun, J. & Salje, E. K. H. Ferroelectric switching in ferroelastic materials with rough surfaces. *Sci. Reports* **9**, DOI: 10.1038/s41598-019-52240-3 (2019).
11. Lu, G., Li, S., Ding, X., Sun, J. & Salje, E. K. H. Tip-induced flexoelectricity, polar vortices, and magnetic moments in ferroelastic materials. *J. Appl. Phys.* **129**, 084104, DOI: 10.1063/5.0039509 (2021).
12. Tyunina, M., Pacherova, O., Kocourek, T. & Dejneka, A. Anisotropic chemical expansion due to oxygen vacancies in perovskite films. *Sci. Reports* **11**, DOI: 10.1038/s41598-021-93968-1 (2021).
13. Duncan, K. L., Wang, Y., Bishop, S. R., Ebrahimi, F. & Wachsman, E. D. Role of point defects in the physical properties of fluorite oxides. *J. Am. Ceram. Soc.* **89**, 3162–3166, DOI: 10.1111/j.1551-2916.2006.01193.x (2006).
14. Cordero, F. *et al.* Effect of doping and oxygen vacancies on the octahedral tilt transitions in the $BaCeO_3$ perovskite. *Phys. Rev. B* **82**, DOI: 10.1103/physrevb.82.104102 (2010).
